# Religiosity and Mental Wellbeing Among Members of Majority and Minority Religions: Findings From Understanding Society: the UK Household Longitudinal Study

**DOI:** 10.1093/aje/kwab133

**Published:** 2021-05-11

**Authors:** Ozan Aksoy, David Bann, Meg E Fluharty, Alita Nandi

**Keywords:** mental health, mental wellbeing, religiosity, religious affiliation

## Abstract

It is unclear whether links between religiosity and mental health are found in contexts outside the United States or are causal. We examined differences in mental wellbeing and associations between mental wellbeing and religiosity among the religiously unaffiliated, White and non-White Christians, Muslims of Pakistani, Bangladeshi, and other ethnicities, and other minority ethnoreligious groups. We used 4 waves of Understanding Society: the UK Household Longitudinal Study (2009–2013; *n* = 50,922). We adjusted for potential confounders (including socioeconomic factors and personality) and for household fixed effects to account for household-level unobserved confounding factors. Compared with those with no religious affiliation, Pakistani and Bangladeshi Muslims and members of other minority religions had worse wellbeing (as measured using the Shortened Warwick-Edinburgh Mental Wellbeing Scale and General Health Questionnaire). Higher subjective importance of religion was associated with lower wellbeing according to the General Health Questionnaire; associations were not found with the Shortened Warwick-Edinburgh Mental Wellbeing Scale. More frequent religious service attendance was associated with higher wellbeing; effect sizes were larger for those with religious affiliations. These associations were only partially attenuated by adjustment for potential confounding factors, including household fixed effects. Religious service attendance and/or its secular alternatives may have a role in improving population-wide mental wellbeing.

## Abbreviations

FIML: full information maximum likelihood
GHQ: General Health Questionnaire
SWEMWBS: Shortened Warwick-Edinburgh Mental Wellbeing Scale

***Editor's note:** An invited commentary on this article appears on page 31, and the authors' response is published on page 36.*

Mental health and wellbeing are important to individuals, families, and society. Mental ill health is a leading contributor to the global burden of disease (1), which makes it imperative that we better understand its modifiable determinants. There is increasing awareness that mental health and wellbeing are multidimensional constructs; positive mental wellbeing may be a different construct than mental ill health (2). Across the nondisordered population, higher positive mental wellbeing appears to have protective effects on other important outcomes, such as physical health (3), and socioeconomic outcomes, such as productivity (4). It is therefore important to identify the modifiable determinants of both mental ill health and positive mental wellbeing.

A growing body of literature—largely conducted in the United States (5, 6) and mostly cross-sectional (7, 8)—has suggested that greater religiosity (particularly religious service attendance) is associated with a lower risk of mental ill health and greater subjective wellbeing. Religious attendance may benefit these outcomes through a myriad of mechanisms, including reducing loneliness, increasing social support, and fostering engagement with other community services (7, 9, 10). Conversely, there could be adverse effects, such as feelings of guilt associated with some religious beliefs or ostracization from other secular societal activities. However, interpretation of the existing literature is currently hampered by difficulty in the generalizability of the results—any effect of religiosity on wellbeing outcomes is likely to differ by societal context and religious denomination. Indeed, Christian faith is a prominent part of public and political life in the United States; (11) more research is therefore needed elsewhere, including analyses of other religious groups (5). Because associations between religiosity and outcomes may be due to confounding and/or reverse causality, (12, 13) research using alternative empirical strategies is also required.

We extended the existing literature by examining associations between multiple religiosity measures and wellbeing outcomes in the United Kingdom—a more secular country than the United States (11). We used a large nationally representative household panel with information on religious affiliation, attendance, and the perceived importance of religion. Its ethnically diverse sample contains considerable heterogeneity in these religiosity measures. We hypothesized that greater religious service attendance would benefit wellbeing across Christian and Muslim groups and among members of other minority religions (14) but that average wellbeing would be lower among Muslims and members of other minority religions because of their increased exposure to discrimination (15, 16), socioeconomic disadvantage (17),and higher levels of acculturation stress (17, 18). Finally, we used the household nature of the study to examine within-household differences in religiosity and wellbeing outcomes to account for unobserved confounding at the household level (19). We hypothesized that effects of religious service attendance would be partly but not fully explained by such household-level confounders such as family socioeconomic status and shared cultural determinants of wellbeing.

## METHODS

### Data

Data from Understanding Society: The UK Household Longitudinal Study (hereafter referred to as Understanding Society) were used (20). This is a nationally representative household panel study started in 2009 with over 70,000 individuals in 30,000 households, which included 4,000 households from an ethnic minority boost sample (21). Investigators attempt to interview all study members who were 16 years of age or older yearly. Individuals who reside in the same households as study members are also interviewed. Wellbeing and other sensitive variables are measured using self-administered questionnaires to reduce social desirability bias. Further details and sampling methodology can be found elsewhere (21). All participants consented for use of their anonymized survey information, and data were accessed through the UK Data Service (https://www.ukdataservice.ac.uk/).

The analytic sample included respondents 16 years of age or older who took part in wave 1 (2009–2011) and wave 4 (2012–2014) and had responded to questions on religiosity or wellbeing. We also used data collected in wave 3 (2011–2013) on personality traits and the number of close friends. The final sample comprised approximately 50,000 persons. We used the outcome variables in wave 2 (2010–2012) for robustness checks.

### Religion and wellbeing measures

Participants were asked whether they belonged to any religion and, if so, which one. Because norms and experiences of individuals from different ethnic groups within the same religion differ—as potentially do the long-term consequences (e.g., subsequent socioeconomic outcomes) (22, 23)—we categorized individuals into ethnoreligious groups rather than just religious affiliation groups. To avoid including ethnoreligious groups with very small sample sizes, we identified groups that were substantively meaningful and had sufficiently large sample sizes (24). For example, 95% and 93% of Pakistani and Bangladeshi respondents, respectively, were Muslim, and 64% of Muslims were Pakistani or Bangladeshi. Thus, we distinguished 1) Pakistani and Bangladeshi Muslims and 2) Muslims of other ethnicities. Using this principle across all affiliation and ethnicities resulted in 5 ethnoreligious groups: nonreligious, White Christian, non-White Christian, Pakistani/Bangladeshi Muslim, other Muslim, and any other group (Sikh, Jewish, Buddhist, Hindu, etc.). We additionally examined specifications whereby religion and ethnicity were treated as separate variables.

Religious attendance was measured by asking “How often, if at all, do you attend religious services or meetings?” with responses of weekly, monthly, yearly, never or practically never, or only at weddings, funerals, etc. The importance of religion was captured by asking “how much of a difference would you say religious beliefs make to your life?” with potential responses of “a great,” “some,” “a little,” or “no” difference. Dummy variables were created for service attendance and religious importance categories.

Wellbeing was measured using the Shortened Warwick-Edinburgh Mental Wellbeing Scale (SWEMWBS) (25, 26) and the General Health Questionnaire (GHQ) (27, 28). SWEMWBS captures positive mental wellbeing in a unidimensional construct with 7 questions on participants’ feeling and thoughts in the past 2 weeks, such as “I’ve been feeling optimistic about the future” and “I’ve been feeling close to other people,” with responses on a Likert scale ranging from “none of the time” to “all the time” (scores range from 7–35, with higher scores indicating better wellbeing). The GHQ is an affective or experienced measure of wellbeing/mental health that captures anxiety, stress, and depressive symptoms. It includes 12 questions that ask how a person felt recently on a 4-point Likert scale. Items capture information on concentration problems, sleep concerns, and difficulty in decision making (scores range from 0–36; we reverse coded the scale so higher scores indicated better mental health).

### Potential confounders

The following were considered as potential confounders: age, gender (male or female; there was no evidence for religiosity × gender interaction), country of birth (England, Scotland, Wales, Northern Ireland, or outside of the United Kingdom), marital status, region (12 categories according to Nomenclature of Territorial Units for Statistics 1 groupings), education (degree, other degree, A-levels, General Certificate of Secondary Education, other qualification, or no qualification), employment status (employed, unemployed, retired, student, at home, long-term sick, or other), natural logarithm of net personal income in pounds, and of the number of close friends + 1, self-rated general health, whether the respondent was ever diagnosed with clinical depression, interaction frequency with neighbors, and personality. All confounders were measured in wave 1 except close friends and personality, which were measured in wave 3.

### Analytical strategy

Associations between religiosity in wave 1 and wellbeing outcomes in wave 4 were examined using linear regressions (results were similar when using the available outcome at wave 2; GHQ only). Because some potential confounders (e.g., income, self-rated health) may operate as mediators, sequential adjustments were made to aid interpretation. Models were first fitted only with the 3 religion variables and then adjusted for 1) potential confounders (age, sex, country of birth); 2) those variables plus outcome measured in wave 1 and clinical depression; and 3) all previous variables and potential factors that may either confound or mediate the observed associations (self-rated health, marital status, income, region, personality, educational level, number of friends, employment status, and communication with neighbors). Models were fitted using full information maximum likelihood (FIML) estimation to reduce the impact of missing data on power and potential bias. Because we also addressed panel attrition with FIML estimation, we applied cross-sectional survey weights in wave 1 and adjusted the standard errors per complex sampling (strata and primary sampling unit).

We then used multilevel models to control for household fixed effects (19); these models accounted for unobserved household-level confounders, such as socioeconomic or cultural factors (formula and illustrative syntax shown in Web Appendix 1, available at https://doi.org/10.1093/aje/kwab133), using *xtreg* in Stata (StataCorp LP, College Station, Texas). Such models estimate the differences in wellbeing outcomes of more religious persons in each household compared with household average, utilizing within-household differences in exposure (42% and 46% of the variation in religious attendance and importance, respectively, are within households when these variables are treated as continuous). Cluster-robust standard errors and wave-1 cross-sectional household-level survey data were used in these models. Because household members are generally of the same ethnoreligious affiliation, household fixed-effects analyses were not used when examining ethnoreligious affiliation (e.g., among 2,208 households with at least 1 Muslim member, only 117 (5%) had a member of another religion). A total of 13,805 single-member households were excluded from the fixed-effects analyses because they had no within-household variations in exposure and outcome.

### Additional and sensitivity analyses

To examine whether wellbeing outcomes were comparable, we checked for measurement invariance across religious affiliation groups and identified potentially problematic items. The main analyses were then repeated with these items removed. We next repeated the main analyses using self-reported life satisfaction (ranging from 0–7) as a cognitive outcome measure. We again repeated analyses and treated religious affiliation (nonreligious, Christian, Muslim, any other) and ethnicity (12 categories) as separate variables instead of combining them into ethnoreligious groups. To examine whether mean differences in outcomes by religiosity was due to differences in the lower or upper tails of the wellbeing distributions, we fitted quantile regression models. To address potential collinearity between ethnoreligious affiliation, attendance, and importance, we added them separately in the models. Finally, we performed a multilevel analysis with random (instead of fixed) intercepts for households.

## RESULTS

**Figure 1:**
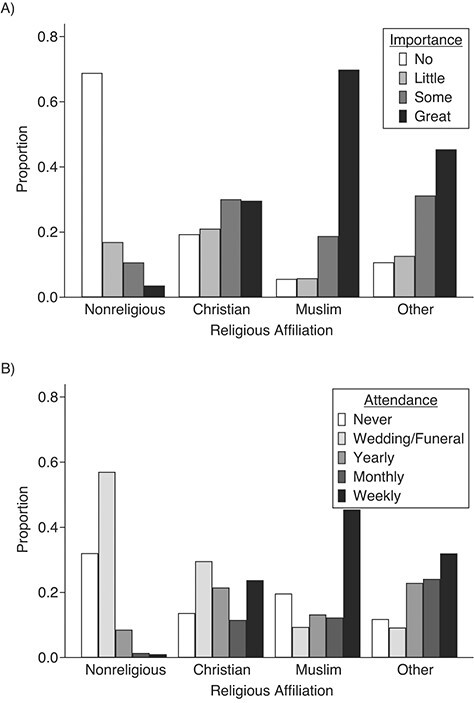
Distributions of A) perceived importance of religion and B) religious service attendance, by religious affiliation, Understanding Society: the UK Household Longitudinal Study wave 1, United Kingdom, 2009–2011.

Of approximately 50,000 wave-1 respondents, 25,114 participants provided complete data for religious affiliation, importance of religion, service attendance, and mental health/wellbeing outcomes in wave 4, and 50,922 participants had nonmissing data for either the SWEMWBS or GHQ score or for at least 1 of the exposure variables and therefore were included in the FIML estimation. See Table 1 for sample sizes for each variable. GHQ and SWEMWBS scores were strongly correlated (0.65 in wave 4 and 0.61 in wave 1). As anticipated, Christians or Muslims reported higher importance of religion and religious attendance than did nonreligious participants (Figure 1). Muslims were more likely than Christians to report religion as being important and to regularly attend religious services (Figure 1). Using SWEMWBS scores, R^2^ values for our models were 1.15%, 2.14%, 28.09%, and 35.04% for religiosity variables, minimal controls, baseline mental health, and all covariates, respectively; using GHQ scores, they were 0.60%, 2.30%, 23.69%, and 30.18%.

**Table 1:** Descriptive Statistics of the Study Sample, Understanding Society: the UK Household Longitudinal Study Waves 1 (2009–2011) and 4 (2012–2014)^a^

**Characteristic**	**No**	**Nonreligious**		**Christian**		**Muslim**		**Other**		***P* Value** ^**b**^
		**%**	**Mean (SD)**	**%**	**Mean (SD)**	**%**	**Mean (SD)**	**%**	**Mean (SD)**	
Religious affiliation	47,659	43.6		43.4		7.9		5.1		
Age, years	47,659		42.1 (16.8)		52.1 (18.3)		35.6 (13.9)		42.5 (16.6)	<0.001
Female sex	47,658	51.2		61.5		52.3		54.1		<0.001
Born outside the United Kingdom	47,651	8.8		15.1		69.3		60.1		<0.001
White British race	47,635	88.5		81.2		5.0		22.6		<0.001
Self-rated health of fair or poor	47,610	20.3		23.5		21.3		22.3		<0.001
Income, pounds (logged)	44,311		6.8 (1.1)		6.8 (1.0)		6.6 (1.1)		6.7 (1.1)	<0.001
Educational level of a degree or higher	47,639	21.1		20.3		23.6		35.4		<0.001
Married	47,642	42.2		56.0		61.5		61.1		<0.001
Neuroticism score	27,183		3.6 (1.5)		3.5 (1.5)		3.6 (1.3)		3.5 (1.4)	0.007
No. of close friends	30,306		5.0 (4.8)		5.4 (5.9)		4.0 (4.9)		4.8 (5.9)	<0.001
Wellbeing										
GHQ score in wave 1	39,665		24.9 (5.3)		25.0 (5.2)		24.9 (5.9)		24.9 (5.7)	0.80
GHQ score in wave 4	25,236		24.9 (5.6)		25.1 (5.4)		24.1 (6.2)		24.8 (5.7)	<0.001
SWEMWBS score in wave 1	38,361		24.9 (4.5)		25.5 (4.5)		24.6 (5.3)		25.5 (4.6)	<0.001
SWEMWBS score in wave 4	25,336		24.4 (4.5)		25.0 (4.4)		23.5 (5.1)		24.7 (4.8)	<0.001

Abbreviations: GHQ, General Health Questionnaire, SWEMWBS, Shortened Warwick-Edinburgh Mental Wellbeing Scale.

^a^ Categorical variables are shown in binary form to aid presentation (all categories were used in regression analyses).

^b^  *P* values were calculated using analysis of variance (for continuous variables) or χ^2^ tests (for categorical variables).

### Religious affiliation and mental health/wellbeing outcomes

**Figure 2:**
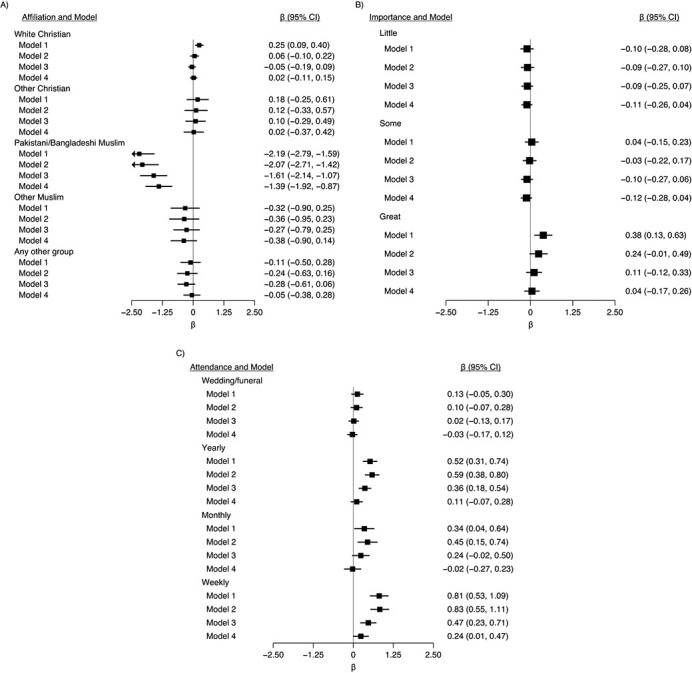
Associations between religiosity measures in wave 1 (2009–2011) and Shortened Warwick-Edinburgh Mental Wellbeing Scale (SWEMWBS) score in wave 4 (2012–2014), Understanding Society: the UK Household Longitudinal Study, United Kingdom. A) Religious affiliation (reference category is nonreligious); B) importance of religion (reference category is none); and C) religious attendance (reference category is never). The total number was 50,922 (72 observations from the original sample of 50,994 were dropped due to the sampling design because 6 strata contained no population members). Model 1 was adjusted only for religion variables; model 2 was additionally adjusted for age, sex, and country of birth; model 3 was additionally adjusted for outcome and clinical depression at baseline (wave 1); and model 4 was additionally adjusted for all other covariates. Full information maximum likelihood estimation was used to account for missing exposure and confounder data, and higher SWEMWBS scores equated to more favorable wellbeing. CI, confidence interval.

**Figure 3:**
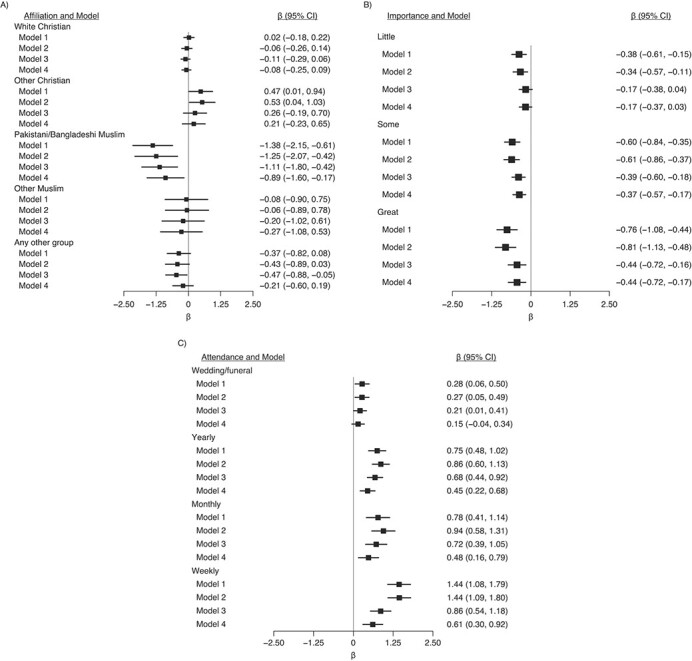
Associations between religiosity measures in wave 1 (2009–2011) and General Health Questionnaire (GHQ) score in wave 4 (2012–2014), Understanding Society: the UK Household Longitudinal Study, United Kingdom. A) Religious affiliation (reference category is nonreligious); B) importance of religion (reference category is none); and C) religious attendance (reference category is never). The total number was 50,922 (72 observations from the original sample of 50,994 were dropped due to the sampling design because 6 strata contained no population members). Model 1 was adjusted only for religion variables; model 2 was additionally adjusted for age, sex, and country of birth; model 3 was additionally adjusted for outcome and clinical depression at baseline (wave 1); and model 4 was additionally adjusted for all other covariates. Full information maximum likelihood estimation was used to account for missing exposure and confounder data. GHQ scores were reverse coded so that higher scores equated to more favorable wellbeing. CI, confidence interval.

Muslims had worse wellbeing which was largely accounted for by the likely colinear variable ethnicity (Web Figure 1). Compared with all participants with no religious affiliation, Pakistani and Bangladeshi Muslims had worse wellbeing outcomes according to both the SWEMWBS and GHQ, and members of other minority groups had worse wellbeing according to GHQ (Figures 2 and 3). These associations were partly attenuated after adjustment for potential confounders; however, associations for Pakistani and Bangladeshi Muslims remained. There was some evidence that Christians (White and other) had higher wellbeing than did those without affiliations; these differences were attenuated to null after adjustment for potential confounders (Figures 2 and 3).

### Importance of religion and mental health/wellbeing outcomes

**Figure 4:**
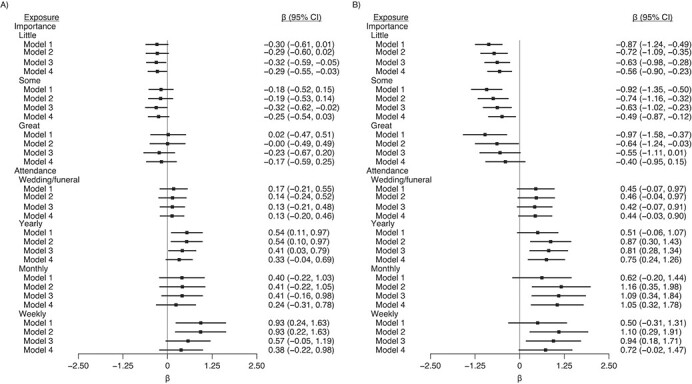
Associations between religiosity measures in wave 1 (2009–2011) and mental wellbeing outcomes health outcomes in wave 4 (2012–2014), Understanding Society: the UK Household Longitudinal Study, United Kingdom. A) Shortened Warwick-Edinburgh Mental Wellbeing (SWEMWBS) scores (*n* = 18,641); B) General Health Questionnaire (GHQ) score (*n* = 18,589), accounting for household fixed effects. Model 1 was adjusted only for religion variables; model 2 was additionally adjusted for age, sex, and country of birth; model 3 was additionally adjusted for outcome and clinical depression at baseline (wave 1); and model 4 was additionally adjusted for all other covariates. The reference categories were as follows: nonreligious for religious affiliation; none for importance; and never for attendance. Higher SWEMWBS and reverse-coded GHQ scores indicate more favorable wellbeing. CI, confidence interval.

Higher reported importance of religion was associated with higher SWEMWBS scores but lower GHQ scores (Figures 2 and 3). The association with SWEMWBS score was attenuated to null once a minimal set of confounders were accounted for; when household fixed effects were accounted for, the association switched sign, but the 95% confidence intervals included the null (Figure 4). The association with GHQ (worse mental health) remained even after controlling for all potential confounders and mediators (Figure 3) and household fixed effects (Figure 4).

### Religious service attendance and mental health/wellbeing outcomes

Attendance was favorably associated with both mental wellbeing outcomes (Figures 2 and 3). For example, compared with participants who never attended religious services, those who attended services weekly had SWEMWBS scores that were 0.81 points (95% CI: 0.53, 1.09) higher and GHQ scores that were 1.44 points (95% CI: 1.08, 1.79) higher (Figures 2 and 3). These changes correspond to approximately 18% and 26% of the standard deviations in SWEMWBS and GHQ scores, respectively (Table 1). These differences were still found, albeit partly attenuated, after adjustment for potential confounders and mediators (Figures 2 and 3) and household fixed effects (Figure 4). Although those who attended weekly had the highest wellbeing scores, the difference between those who attended monthly and those who attended yearly was small.

**Figure 5:**
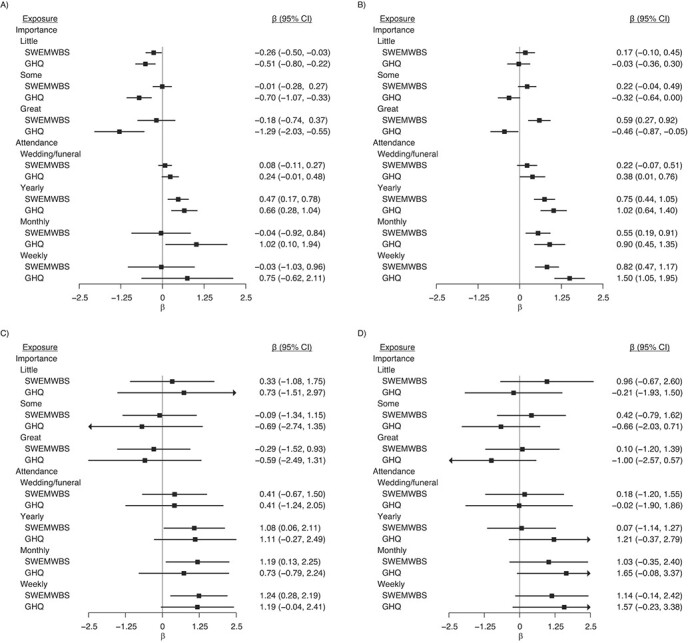
Associations between importance of religion and religious attendance in wave 1 (2009–2011) and mental wellbeing outcomes in wave 4 (2012–2014) estimated with full information maximum likelihood estimation, Understanding Society: the UK Household Longitudinal Study, United Kingdom. A) Nonreligious participants (*n* = 20,502); B) Christians (*n* = 20,565); C) Muslims (*n* = 3,742); and D) members of other minority religions (*n* = 2,438). The model was adjusted for sex, age, and country of birth. Higher SWEMWBS and reverse-coded GHQ scores indicate more favorable wellbeing. The reference categories were as follows: nonreligious for religious affiliation; none for importance; and never for attendance. CI, confidence interval.

We found suggestive evidence that the associations between religiosity and outcomes were more positive for those with religious affiliations (Figure 5); however, confidence intervals in each group overlapped (*P* for affiliation × attendance = 0.702 for SWEMWBS and 0.801 for GHQ; *P* for affiliation × importance = 0.020 for SWEMWBS and 0.094 for GHQ). There was no evidence for interaction when restricted to those with religious affiliations (*P* (affiliation × attendance = 0.499 for SWEMWBS and 0.873 for GHQ), *P* (affiliation x importance = 0.168 for SWEMWBS and 0.941 for GHQ)).

### Additional and sensitivity analyses

Findings were similar when 1) excluding items that lowered psychometric invariance of SWEMWBS and GHQ scores (Web Appendix 2); 2) using life satisfaction (Web Figure 2); 3) entering the 3 religious variables separately (Web Figure 3); and 4) using random household effects (Web Figure 4). The average differences reported above (Figures 2–4) were driven particularly by differences at the most negative parts of the mental health/wellbeing distribution (Web Figure 5).

## DISCUSSION

### Main findings

Using nationally representative household data from the United Kingdom, we found that Muslims had lower average mental wellbeing scores than did Christians or those with no religious affiliation. More frequent religious service attendance was associated with higher mental wellbeing. This association was found across 2 outcomes and was stronger for those with religious affiliations. These findings were robust to adjustment for multiple confounders and after accounting for unobserved household-level confounders. In contrast, the subjective importance of religion was not associated with higher mental wellbeing; those who reported greater importance of religion on life in fact reported more mental health symptoms.

### Comparisons with previous evidence and explanation of findings

Our findings are consistent with previous evidence—largely conducted in the United States on samples of Christians—that suggested that there are beneficial effects of religious attendance (5–8). They are also consistent with the only randomized controlled trial of which we are aware, which suggested causal effects of religiosity (29). However, given the specific intervention (evangelical Christianity) and target population (low-income Filipino households), generalizing from this ethically contentious trial is challenging. The findings are also consistent with those from a natural experimental study, which suggested that greater involvement in a religious activity (Ramadan fasting) increases wellbeing among Muslims (14).

We observed notable differences in wellbeing outcomes according to ethnoreligious group, which reflects the effects of both religious affiliation and ethnicity. Recent research has shown that globally, Christians appear to be happier than both those without religious affiliations and Muslims (30, 31). In our analyses, however, there was no evidence that Christians had higher wellbeing than those without affiliation after accounting for a minimal set of confounders (notably age). Any association between religious affiliation and wellbeing might therefore be context dependent. In contrast, there was consistent evidence—before and after adjustment for confounders—that the Pakistani/Bangladeshi Muslims had worse wellbeing than did those without affiliation. The negative associations between belonging to a minority ethnoreligious group and wellbeing may reflect harassment and discrimination (15, 32), socioeconomic disadvantage (17), and higher levels of acculturation stress (17, 18). Larger samples with variation across religious affiliation, ethnicity, and socioeconomic disadvantage are likely required to attempt to separate out effects due to religious affiliation and ethnicity. Religious service attendance, however, was positively associated with wellbeing among members of minority religions. This suggests that, consistent with results from past research, service attendance may buffer the negative consequences of belonging to a minority religion (33).

After using longitudinal data and factoring in household fixed effects to account for unobserved household level confounders, our results are consistent with there being a positive causal effect of religious service attendance as opposed to subjective religious beliefs on mental wellbeing (9). The effect of service attendance may operate via multiple mechanisms that may differ depending on the religion and societal context. These mechanisms include direct and indirect impacts of social networks, such as providing social support, reducing loneliness, and fostering engagement with other community services (9).

We found that reported importance of religion had either no or potentially negative associations with wellbeing. This finding may reflect acculturation, guilt associated with some religious beliefs, and/or unobserved factors that select into both worse wellbeing and greater perceived importance of religion.

Despite our use of longitudinal data and accounting for multiple potential confounders and household fixed effects, our findings may still reflect noncausal relationships. First, findings may reflect reverse causality—mental ill health may impede attendance in religious activities. Although we used longitudinal data that was adjusted for baseline mental ill health/wellbeing scores, there may be remaining residual impacts of previous mental health on religious attendance. Reverse causality may also have an effect on analyses within households (fixed-effect analysis); however, this method is likely to better account for household-level invariant confounding factors, such as family socioeconomic status. Ultimately, given the practical and ethical barriers to using randomized trials for this topic—and difficulties in generalization from trials—future progress will likely be guided by findings from observational studies (34).

### Strengths and limitations

Our study was limited by a relatively short follow-up period (approximately 3 years). Thus, longer follow-up is required, given concerns over reverse causality. It is possible, however, that causal beneficial effects of religious attendance in the United Kingdom are in fact short term and thus weak or nonexistent when using longer periods of follow-up. Nevertheless, evidence in the United States suggests that associations are indeed observed across longer time periods (35). The outcome variables in wave 4 were missing for approximately half of the wave-1 sample. This was addressed using FIML estimation, which produces unbiased estimates if missingness depends on observed data only (i.e., missing at random) and under multivariate normality (36). Because we controlled several key variables in the analysis, violations of missing at random should arguably be minor. Moreover, results with listwise deletion were similar to FIML estimates.

Strengths of the study include the large sample from a variable population, which enabled examination of Muslims, a previously underrepresented group in studies of religion and wellbeing. Indeed, religion is noted stratifier of health inequality according to World Health Organization guidance (37). However, there is substantial within-group heterogeneity in each religion regarding religious belief and practice that we were unable to investigate. Although we used a large nationally representative study with considerable religious heterogeneity, we were underpowered to investigate wellbeing outcomes in smaller religious groups. This warrants investigation with more granular data on religious affiliation.

Although residual confounding cannot be ruled out, our analyses contained substantial data on potential confounders that were unavailable in much prior research; further, we utilized household fixed-effects analyses. We also considered several outcomes and found similar findings across them. Unlike existing studies in which the focus was on mean differences only, we also used quantile regression and found that mean differences were driven by those with poorer mental health.

### Potential implications

If associations between religious service attendance and outcomes are causal, our findings may have implications for strategies to improve population-wide mental health. Given the increasing levels of mental ill health observed in the population (38) and the decline in religious attendance observed in the West, (11) one naive suggestion would be that religious service attendance should be increased across the entire population. However, we would caution against such suggestions because alignment is clearly required between individuals’ faith and the religious services available. Further, there may be other deleterious consequences of such attendance that we do not observe (13, 39). Indeed, we found that associations between religious service attendance and positive wellbeing outcomes were most evident among those with religious affiliations, although we were unlikely powered to detect differences between each subgroup. Instead, we argue for a need for secular alternatives to religious services that can replicate and/or improve upon their potential benefits, regardless of religious faith. Many predominant religious institutions have benefited from centuries of publicly subsidized development, resulting in established physical and social capital. Secular alternatives to religious services, such as the Sunday Assembly (established in 2013), and organizations that seek (from a secular perspective) to improve society and aid individuals, such as Humanists UK and the American Humanist Association, are arguably in their infancy. Other civic organizations without such explicit goals may confer benefits similar to those gained from religious institutions. Indeed, one potential explanation of the worsening of mental health outcomes in recent decades (38) is the increasing individualized nature of society, which is characterized by declines in communal activities like religious service attendance (40).

Among those with religious faith, our findings may suggest that facilitating religious service attendance may be one means by which the negative consequences of belonging to a minority religion could be averted. Such considerations may particularly benefit already vulnerable groups—for example, data from a recent report implied that in the United Kingdom, although most masjids and mosques have facilities for women, those facilities are typically limited in smaller masjids and in some ethnic minority groups (41).

### Conclusions

Associations between religious service attendance and mental wellbeing were found in the United Kingdom and were present for both majority (Christianity) and minority religions. Our longitudinal and household fixed-effects analyses support the notion that such associations may be causal. In contrast, we found a strong negative association between belonging to a minority ethnoreligious group and mental wellbeing. Religious service attendance and/or its secular alternatives may have a role in improving population-wide mental wellbeing.

## Supplementary Material

Web_Material_kwab133
