# Supplementary material for: Religiosity and Mental Wellbeing Among Members of Majority and Minority Religions: Findings From Understanding Society: the UK Household Longitudinal Study
Source: Am J Epidemiol. 2021 May 11;191(1):20–30. doi: 10.1093/aje/kwab133 (PMC8751808; doi:10.1093/aje/kwab133)
Supplement: Web_Material_kwab133 [file web_material_kwab133.pdf]

Web material to “Religiosity and mental wellbeing among members of majority and minority religions: findings from Understanding Society, The UK Household Longitudinal Study”

Ozan Aksoy, David Bann, Meg E Fluharty, Alita Nandi

Contents

Web Appendix 1. Fixed-effect specification 2

Web Appendix 2. Testing measurement invariance of SWEMWBS and GHQ across religions 3

Web Figure 1. Associations between religious affiliation (whereby religious affiliation and ethnicity are treated as separate variables), religiosity measures, and mental wellbeing outcomes. 7

Web Figure 2. Associations between religiosity measures and life satisfaction (range from 0 (lowest) to 7 (highest). 8

Web Figure 3. Associations between the three religiosity measures (ethnoreligious groups, religious attendance and importance) and mental wellbeing outcomes health outcomes when the three variables are entered in the model separately. 9

Web Figure 4. Associations between ethnoreligious groups, religious attendance and importance with mental wellbeing outcomes when household random (rather than fixed) intercepts are included in models. 10

Web Figure 5. Associations between religiosity measures and mental wellbeing outcomes in quantile regressions. 11

Web Appendix 1. Fixed-effect specification

Equation for fixed effects analysis within households:

$$y_{ih} = \beta_0 + X_{ih}\beta + \alpha_h + u_{ih}$$

Where:

- *y* is the outcome for each *i* individual within *h* household.
- X_{ih} is a vector of household-variable exposures.
- α_h are household-level fixed effects that adjust for all time constant household level factors, such as socioeconomic factors.
- u_{ih} is the individual-level residual term.

In Stata, the above can be estimated using *xtreg*, for example:
xtreg outcome exposure [pweight = hholdweight], i(household_id) fe cluster(household_id)

Web Appendix 2. Testing measurement invariance of SWEMWBS and GHQ across religions

SWEMWBS

Here, we test measurement invariance of the 7-item version of the Warwick-Edinburgh mental well-being scale across three religion categories: the non-religious, Christians of any denomination, and Muslims of any denomination. We exclude members of other religions from the analysis. We use the data from Understanding Society Wave 1. The seven items are (1) feeling optimistic about the future (2) feeling useful (3) feeling relaxed (4) dealing with problems well (5) thinking clearly (6) feeling close to others (7) able to make up own mind. A graphical representation of the measurement model is given below.

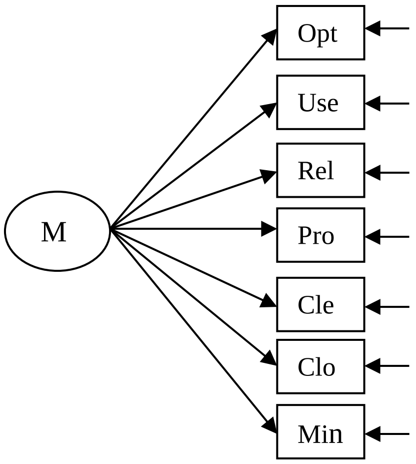

We fit four models with varying degrees of strictness of measurement invariance (see e.g. Kline 2016). The first one is the *configual invariance* model, which keeps the model structure in the above depiction the same, but allows the parameters to vary across the three groups. The second is the *weak invariance* model, which in addition to the configural invariance, constrains the loadings of the items on the latent mental wellbeing factor to be the same in the three groups. The third is a *strong invariance* model which constrains, in addition to the loadings, the intercepts of items to be the same across the three groups. Finally the *strict invariance* model constrains loadings, intercepts, and the error variances of the items.

Web Table 1 below shows a number of fit measures for the four models. Firstly, the exact fit hypothesis for the configural invariance is rejected. This implies that the most unconstrained model fits data less than perfect to start with. But also note that N is rather large in our case (36,623) so even small misfits may become insignificant. Our aim is not to test or validate the SWEMWBS scale itself, but establish its invariance across the three groups. So we leave a side the misfit of the configural invariance model. We would like to report, however, that adding a covariance between the error terms of item 1 and 2 and between item 5 and 7 improves the model fit rather significantly. Adding these two error covariances reduces the model $\chi 2$ to 2268.22 (36), RMSEA to 0.071, BIC to 556484.48, SRMR to 0.023 and increases CFI to 0.979. Because invariance of error covariances across the groups is not strictly required for sufficient measurement invariance, we carry on with the original specification without the error covariances. The conclusions reported below are the same if we carry out our analyses with the two error covariances added.

Web Table 1. Fit measures of various measurement invariance models; N = 36,623.

	Configural inv.	Weak inv.	Strong inv.	Strict Inv.
$\chi 2$	6288.96***	6341.98***	6555.05***	6976.70***
(df)	(42)	(54)	(66)	(80)
RMSEA	0.110	0.098	0.090	0.084
(90% CI)	(0.108-0.113)	(0.096-0.100)	(0.088-0.092)	(0.082-0.086)
AIC	559906.14	559935.16	560124.23	560517.88
BIC	560442.17	560369.09	560456.06	560730.59
CFI	0.941	0.940	0.938	0.934
TLI	0.911	0.930	0.941	0.948
SRMR	0.039	0.046	0.052	0.068

The fit of the weak invariance model is comparable to the configural invariance model. While a likelihood ratio test favors the configural invariance over weak invariance (compare the $\chi 2$ values of the two models), this could again be due to very large N. Other fit measures, in fact, indicate that the weak invariance model fits somewhat better than the configural invariance model (e.g. RMSEA, BIC, and TLI). A comparison of item loadings across the three religion groups in the configural invariance model shows that the loadings vary only marginally. The largest differences are for the loadings of item 6 and 7, both of which are somewhat higher among Muslims and Christians than the non-religious. In fact, removing items 6 and 7 from the scale makes the $\chi 2$ difference test between the weak invariance and the configural invariance models statistically insignificant. While there is a case to remove these two items from the model, the sizes of the differences in the loadings across the religion groups seem rather minor.

The conclusions are similar if we compare the strong and strict invariance models with the configural invariance model. We thus conclude that the short version of the Warwick-Edinburgh mental well-being scale measures mental well-being rather similarly across the three religion groups with a caveat regarding item 6 and 7.

GHQ

Now, we test measurement invariance of the 12-item subjective well-being score (GHQ) across the non-religious, Christians of any denomination, and Muslims of any denomination. We will use the data from Understanding Society Wave 1. The 12 items ask about (1) concentration (2) loss of sleep (3) playing a useful role (4) capable of making decisions (5) constantly under strain (6) problem overcoming difficulties (7) enjoy day-to-day activities (8) ability to face problems (9) unhappy or depressed (10) losing confidence (11) believe in self-worth (12) general happiness. The same as above, we fit four models with varying degrees of strictness of measurement invariance (see e.g. Kline 2016), namely *configual invariance, weak invariance strong invariance* and *strict invariance* models.

Web Table 2 below shows the fit measures of these four models. The exact fit hypothesis for the configural invariance is flatly rejected. This implies that the most unconstrained model fits data poorly to start with. This poor fit could be due to very large N, but even other approximate fit measures (CFI, TLI, and SRMR) indicate rather poor fit. This implies that GHQ may not be measuring a one-dimensional concept. A likelihood ratio test rejects the weak invariance model in favour of the configural invariance model ($\chi 2$ (22) = 152.86, $P < 0.001$). This again could be due to very large N. In fact, other fit measures of the weak invariance model show an improvement of model fit compared to the configural invariance model. RMSEA, BIC, TLI, and CIT for example favour the weak invariance model. This shows that measurement invariance of GHQ across the three religions can be accepted, however, the unconstrained model fits rather poorly.

Web Table 2. Fit measures of various measurement invariance models of GHQ; N = 37,868.

	Configural inv.	Weak inv.	Strong inv.	Strict Inv.
χ2	23427.206***	23580.069***	23993.508***	26490.634***
(df)	(162)	(184)	(206)	(230)
RMSEA	0.107	0.100	0.096	0.095
(90% CI)	(0-.)	(0-.)	(0-.)	(0-.)
AIC	697474.898	697583.761	697953.200	700402.325
BIC	698397.419	698318.361	698499.879	700744.000
CFI	0.878	0.877	0.875	0.862
TLI	0.851	0.868	0.880	0.882
SRMR	0.055	0.060	0.064	0.130

We now modify the configural invariance model to attain a relatively better fitting baseline model. After inspecting the R-squared per item and the differences in loadings across the three religions, we remove items 1, 3, 4, 7, and 8. The R-squared values for these items are respectively 33%, 23%, 27%, 36%, and 34%. These values are too low to justify including them in the same scale. Next, looking at modification indices, we add an error covariance between item 11 and item 12 and between item 2 and item 5. This results in a relatively well fitting modified configural invariance model with 7 items. We now test various invariance models building on this modified configural invariance model.

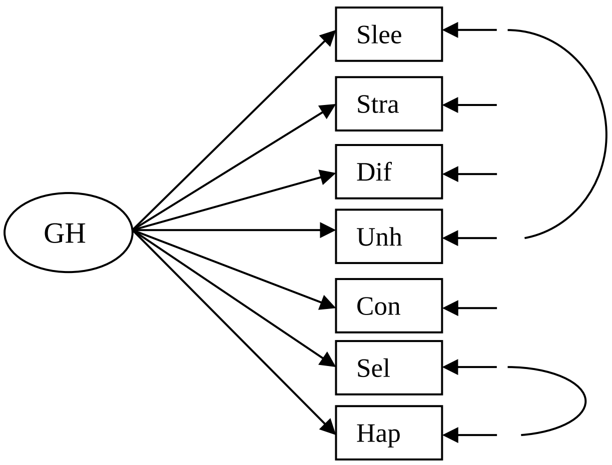

The fit of the modified weak invariance model is comparable to the modified configural invariance model. While a likelihood ratio test favors the configural invariance over weak invariance (compare the χ2 values of the two models), this could again be due to very large N. Other fit measures indicate that the modified weak invariance model fits better than the configural invariance model (e.g. RMSEA, BIC, CFI and TLI). Also, in the modified configural invariance model the loadings differ only marginally across the religions. Strong and strict invariance models also fit data relatively well. We thus conclude that the reduced version of the GHQ scale measures wellbeing relatively similarly across the three religions.

Web Table 3. Fit measures of various measurement invariance models of the modified GHQ; N = 37,868.

	Configural inv.	Weak inv.	Strong inv.	Strict Inv.
χ2	2330.605***	2395.394***	2599.167***	3314.358***
(df)	(36)	(48)	(60)	(78)
RMSEA	0.071	0.062	0.058	0.057
(90% CI)	(0.068-0.073)	(0.060-0.064)	(0.056-0.060)	(0.056-0.059)
AIC	471688.735	471729.524	471909.297	472588.488
BIC	472278.434	472216.667	472293.884	472819.240
CFI	0.981	0.981	0.979	0.973
TLI	0.967	0.975	0.978	0.979
SRMR	0.024	0.028	0.036	0.074

Effects of religion on modified versions of SWEMWBS and GHQ

The Figure below shows the effect of religion variables on the modified, reduced versions of GHQ and of SWEMBWS (items 6 and 7 removed). The models rely on complete-case analysis and original GHQ scale (i.e. not-reverse coded as in the main manuscript), and that for simplicity importance and attendance variables were scaled to the [0 1] range and treated as continuous. These effects are similar to the ones with the full versions of GHQ and SWEMBWS. Note that these effects are estimated with a complete-case analysis, hence standard errors tend to be somewhat larger.

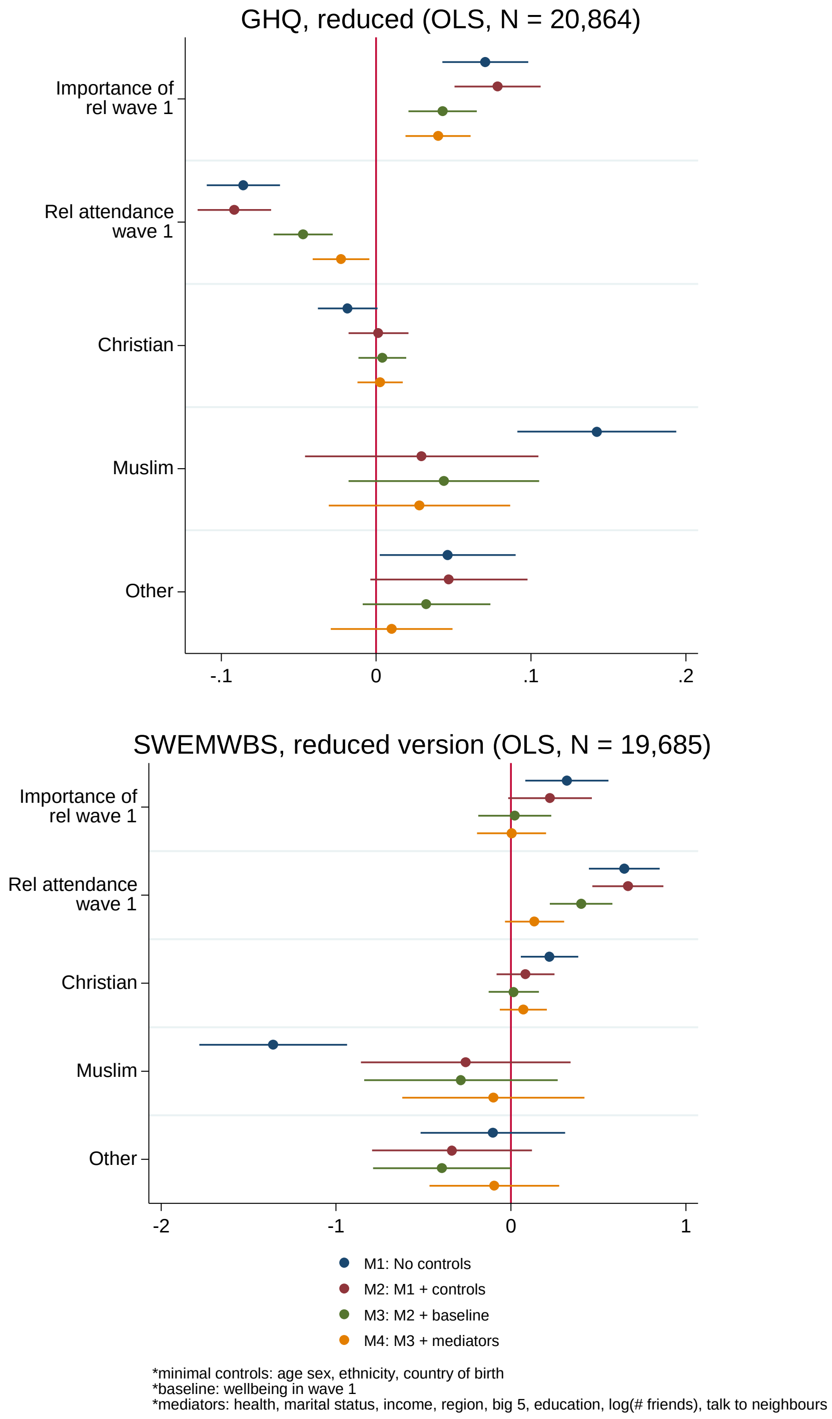

Figure. Religiosity variables in relation to reduced versions of SWEMWBS and GHQ.

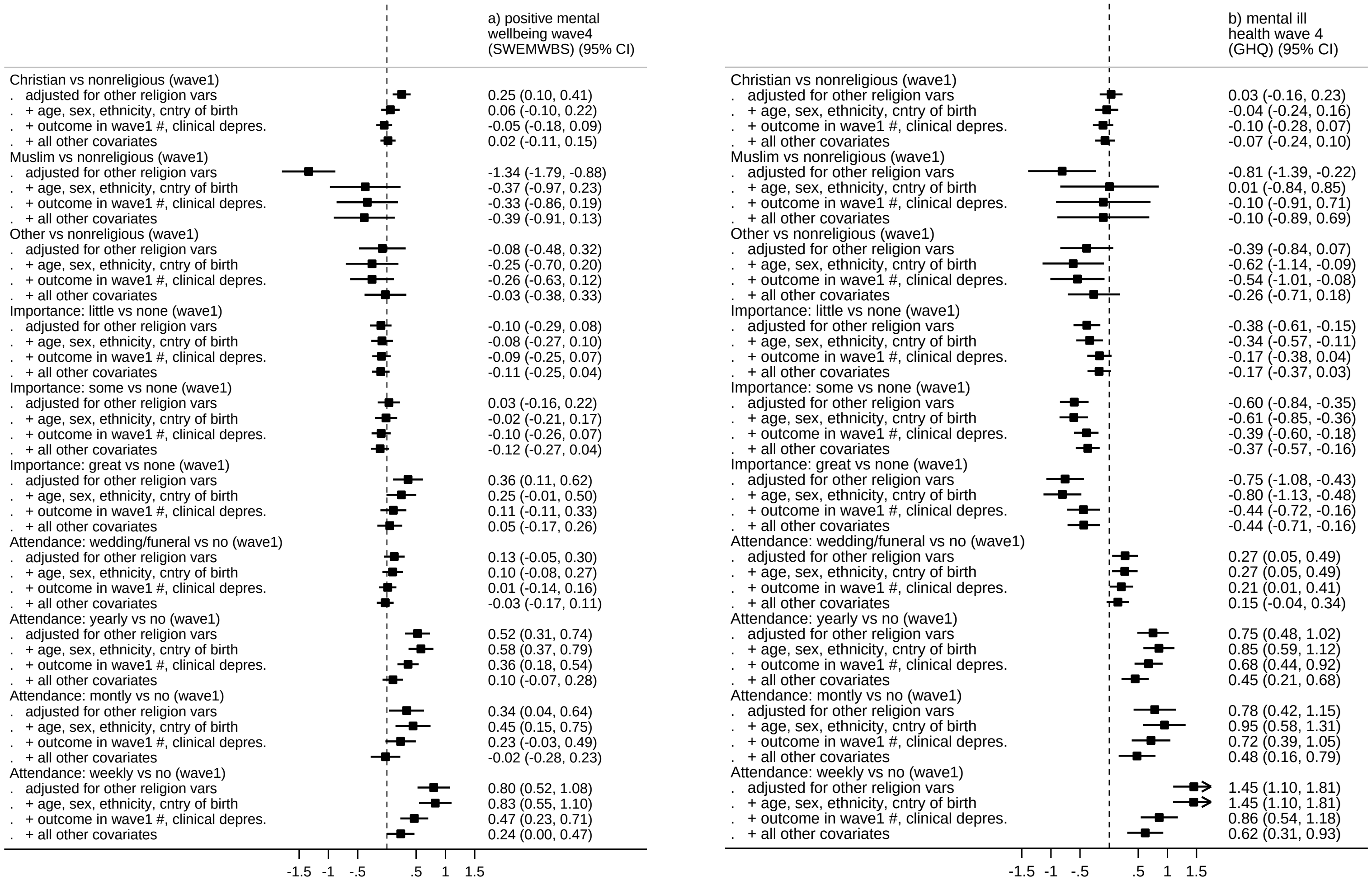

Web Figure 1. Associations between religious affiliation (whereby religious affiliation and ethnicity are treated as separate variables), religiosity measures, and mental wellbeing outcomes.

Note: Wave 1 (2009/2011) and Wave 4 (2012-2014); Full Information Maximum Likelihood Estimation (FIML) was used to account for missing exposure and confounder data.

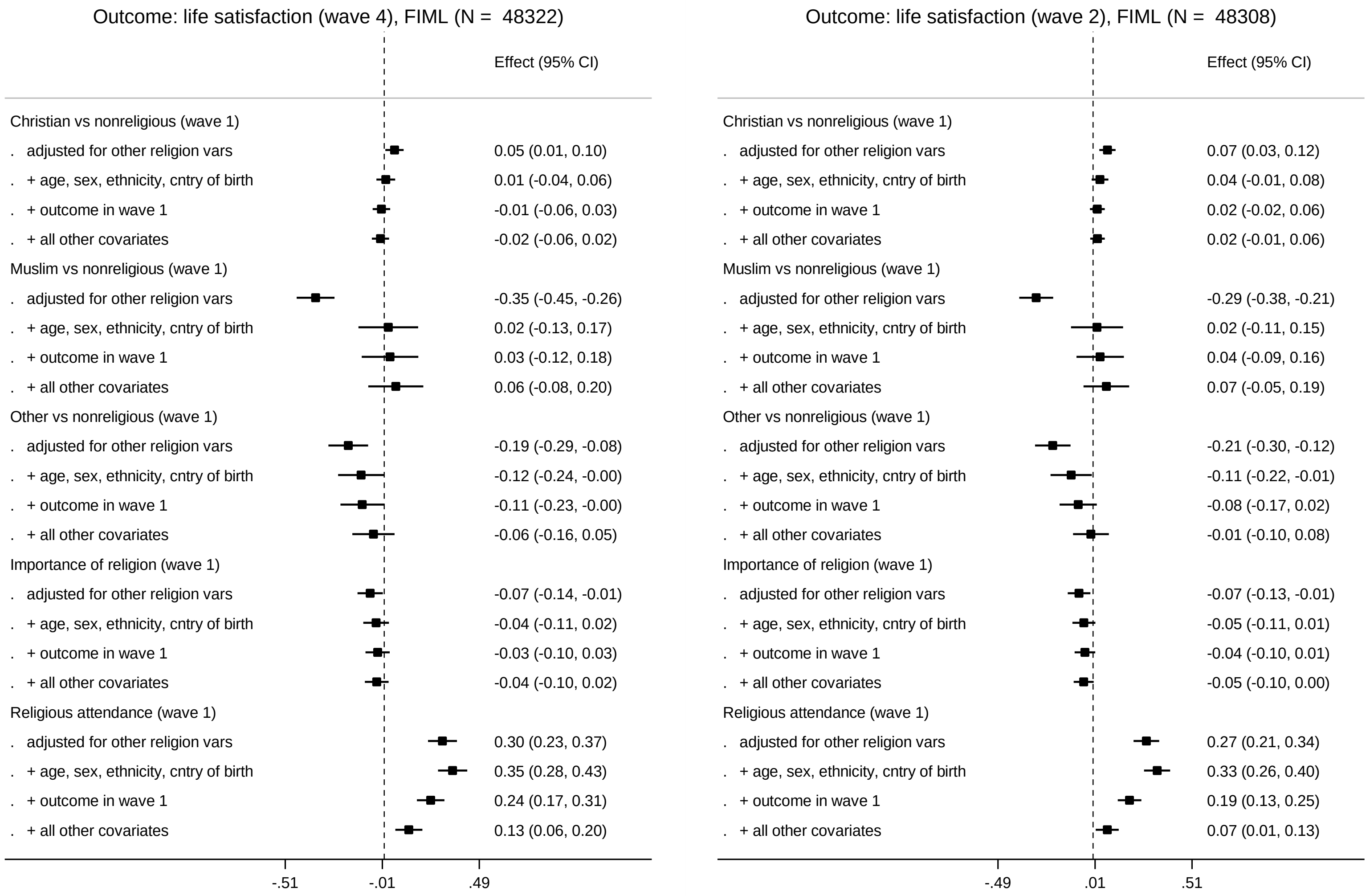

Web Figure 2. Associations between religiosity measures and life satisfaction (range from 0 (lowest) to 7 (highest).

Note: Wave 1 (2009/2011) and Wave 4 (2012-2014); Full Information Maximum Likelihood Estimation (FIML) was used to account for missing exposure and confounder data. To simplify interpretation religious attendance and importance were scaled to the [0 1] range and treated as continuous.

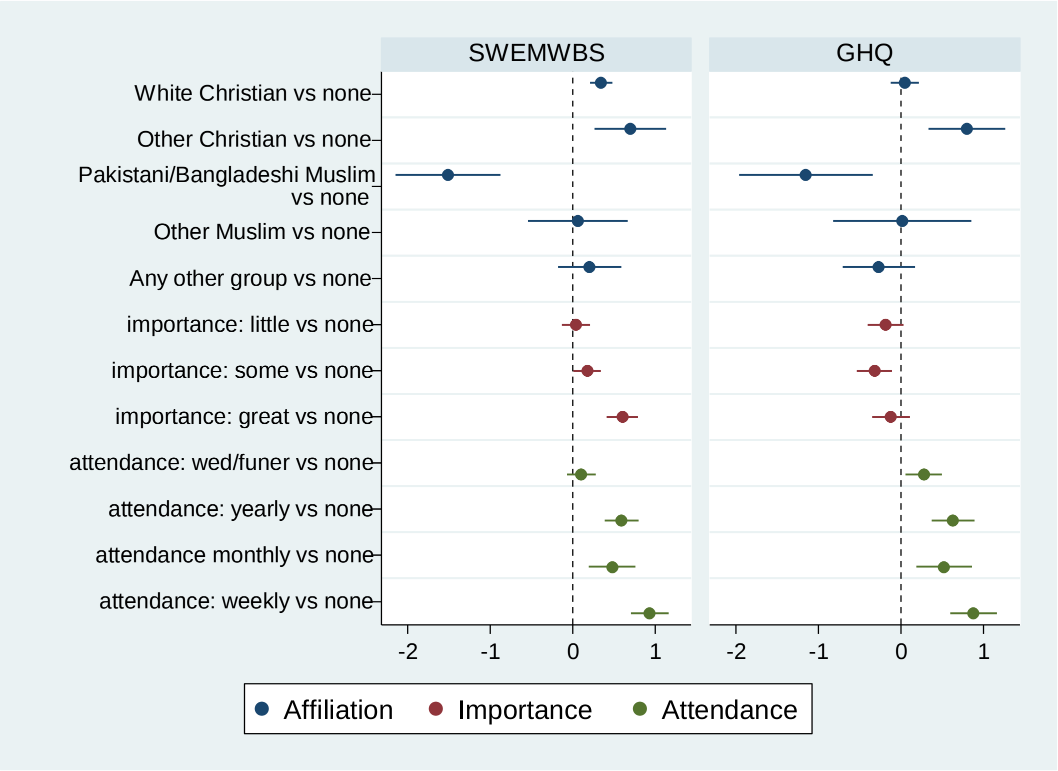

Web Figure 3. Associations between the three religiosity measures (ethnoreligious groups, religious attendance and importance) and mental wellbeing outcomes health outcomes when the three variables are entered in the model separately.

Note: Shortened Warwick-Edinburgh Mental Wellbeing (SWEMWBS), left panel; General Health Questionnaire (GHQ) score, right panel), with the three variables entered separately in the model, adjusted for age, sex, and country of birth. Note: Religiosity measured in Wave 1 (2009/2011) and outcomes in Wave 4 (2012-2014); higher SWEMWBS and GHQ (reverse coded) scores equate to more favourable wellbeing.

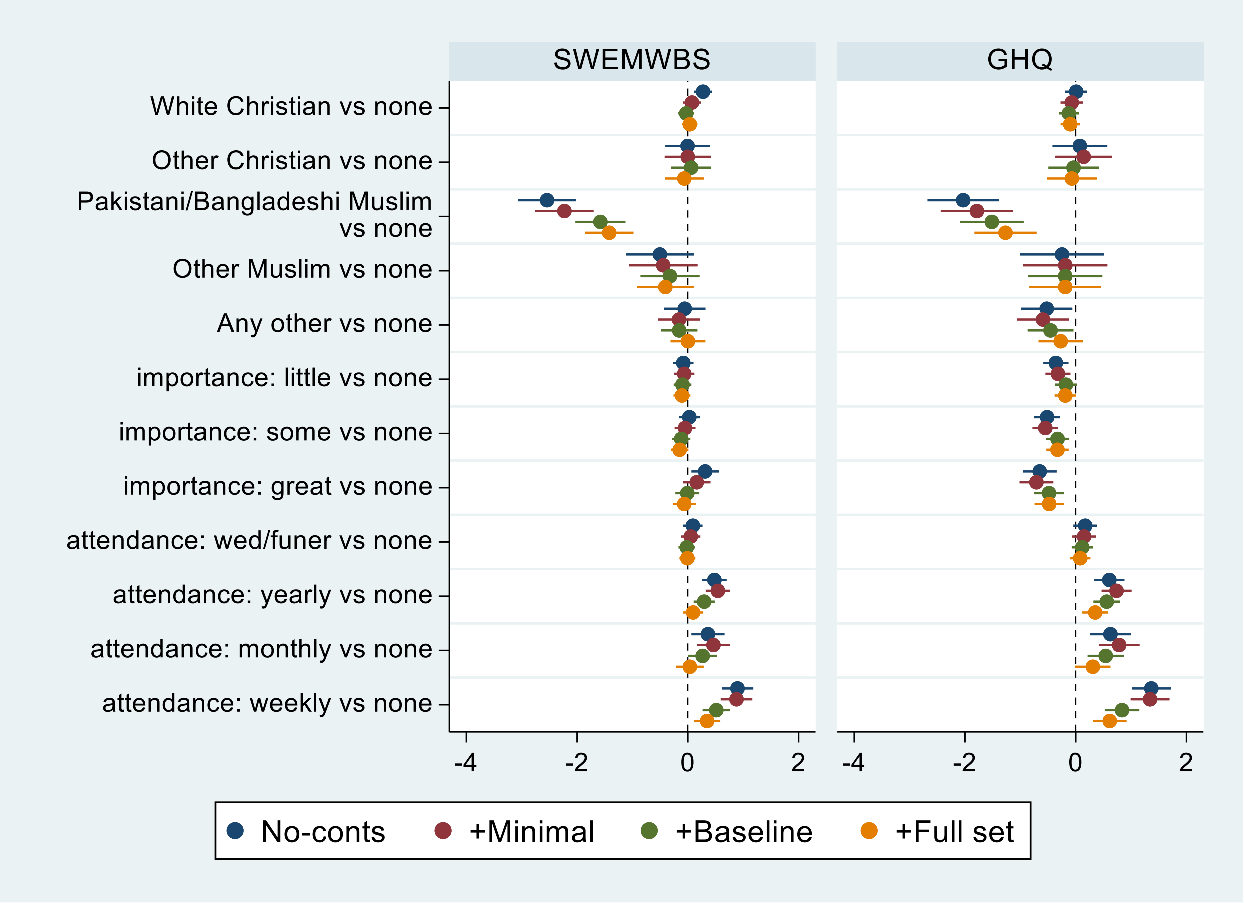

Web Figure 4. Associations between ethnoreligious groups, religious attendance and importance with mental wellbeing outcomes when household random (rather than fixed) intercepts are included in models.

Note: The legend describes the model fitted, viz. No-conts: no additional control variables, +Miminal: minimal set of controls included, + Baseline: additionally wave 1 values of the outcome included, + Full set: additionally all other covariates are included in the model. Estimates are based on complete case analysis. Shortened Warwick-Edinburgh Mental Wellbeing (SWEMWBS), left panel; General Health Questionnaire (GHQ) score, right panel. Religiosity measured in Wave 1 (2009/2011) and outcomes in Wave 4 (2012-2014); higher SWEMWBS and GHQ (reverse coded) scores equate to more favourable wellbeing.

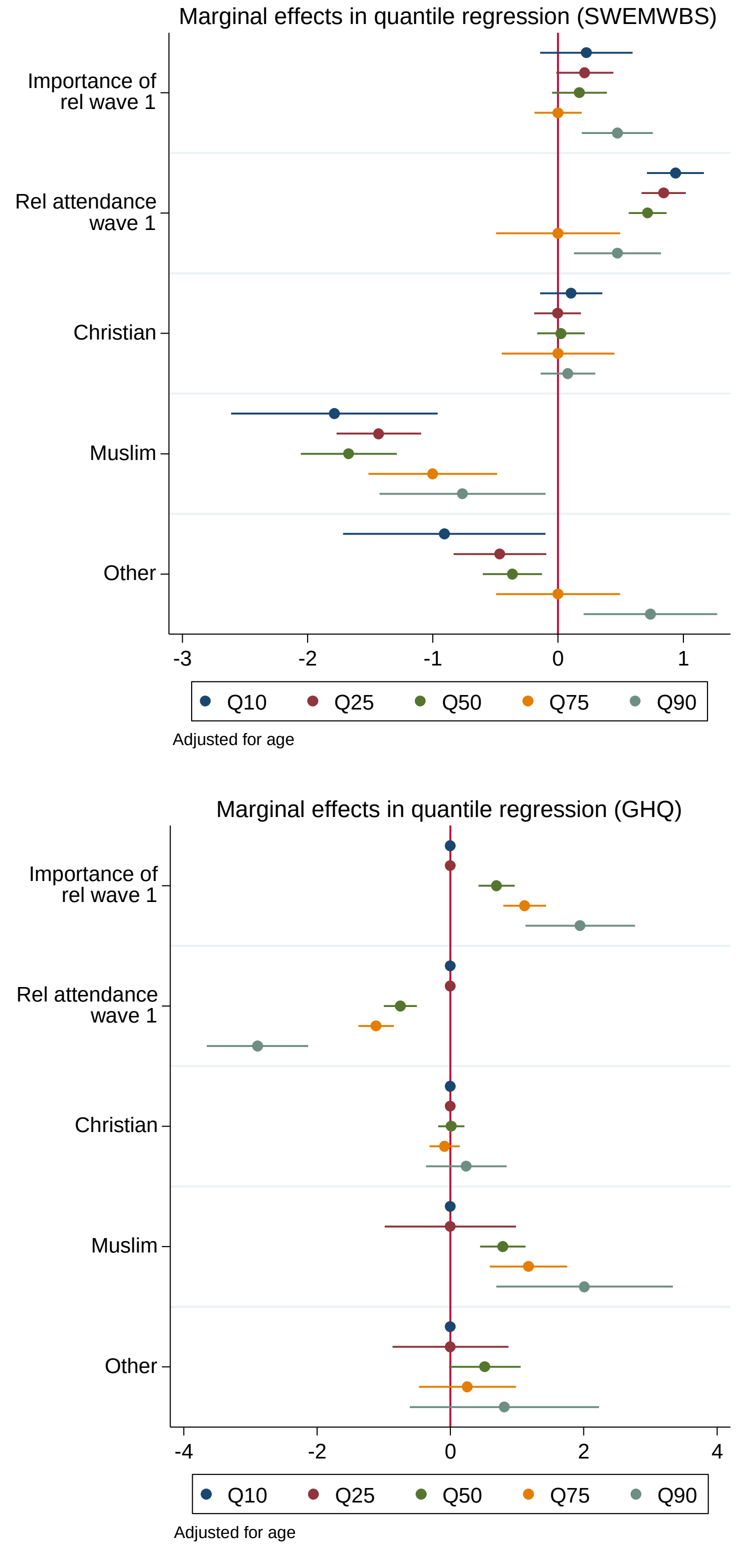

Web Figure 5. Associations between religiosity measures and mental wellbeing outcomes in quantile regressions.

Note: Religiosity measured in Wave 1 (2009/2011) and outcomes in Wave 4 (2012-2014); quantile regression models were used—coefficients are interpreted analogously to linear regression: e.g., Q50 shows the median difference in mental wellbeing comparing those with Muslim compared with no religious affiliation, while Q90 shows the difference at the 90th quantile (far right-end of the distribution). For simplicity religious attendance and importance were scaled to the [0 1] range and treated as continuous.
